# Dietary *Lasia spinosa* Thw. Improves Growth Performance in Broilers

**DOI:** 10.3389/fnut.2021.775223

**Published:** 2022-01-13

**Authors:** Lang Zhang, Yongxing Hong, Yuying Liao, Kui Tian, Haodong Sun, Xingting Liu, Yanfei Tang, Abdallah A. Hassanin, Sameh A. Abdelnour, Wanwipa Suthikrai, Kittiya Srisakwattana, Theerawat Tharasanit, Yangqing Lu

**Affiliations:** ^1^State Key Laboratory for Conservation and Utilization of Subtropical Agro-Bioresources, College of Animal Science and Technology, Guangxi University, Nanning, China; ^2^Guangxi Veterinary Research Institute, Nanning, China; ^3^Guangxi Fufeng Agriculture and Animal Husbandry Co Ltd, Nanning, China; ^4^Faculty of Agriculture, Zagazig University, Zagazig, Egypt; ^5^Research and Development Center for Livestock Production Technology, Faculty of Veterinary Science, Chulalongkorn University, Bangkok, Thailand; ^6^Department of Obstetrics, Gynecology and Reproduction, Faculty of Veterinary Science, Chulalongkorn University, Bangkok, Thailand

**Keywords:** broiler, *Lasia spinosa* Thw., growth performance, blood metabolites, antioxidant status, intestinal morphology, cecal microbiome

## Abstract

This study aimed to evaluate the effects of dietary *Lasia spinosa* Thw. (LST) powder supplementation on growth performance, blood metabolites, antioxidant status, intestinal morphology, and cecal microbiome in broiler chickens. A total of 400 1-day-old male Guangxi partridge broilers (initial body weight: 42.52 ± 0.06 g) were randomly allotted to 4 dietary treatments: LST0 group (a basal diet), LST1 group (a basal diet with 1% LST powder), LST2 group (a basal diet with 2% LST powder), LST4 group (a basal diet with 4% LST powder), 10 replicates for each treatment, and 10 broilers in each treatment group. Results indicated that the average daily feed intake of broilers during 22–42 days and the average daily gain of chickens during 1–42 days significantly increased by dietary supplementation of LST powder (*p* < 0.01), while the feed conversion ratio during the overall periods was decreased by dietary supplementation of LST powder (*p* < 0.01). Except for the levels of superoxide dismutase (SOD) and glutathione peroxidase (GSH-Px) in liver (*p* > 0.05), the levels of SOD, catalase (CAT) and GSH-Px in serum, liver, and breast muscle were significantly increased in the LST supplemented groups (*p* < 0.05), while the levels of reactive oxygen species (ROS) and malondialdehyde (MDA) in serum, liver, and breast muscle were significantly decreased in the LST supplemented groups (*p* < 0.05). Furthermore, the levels of triglyceride (TG) and low-density lipoprotein cholesterol (LDL-C) were significantly decreased by the addition of dietary LST powder (*p* < 0.01), while the levels of HDL-C, Ca, Fe, Mg, and P were linearly increased by the addition of dietary LST powder (*p* < 0.01). With respect to the gut morphometric, crypt depth was significantly decreased by LST supplementation (*p* < 0.05), while villus height and the ratio of villus height to crypt depth were notably increased by LST supplementation (*p* < 0.05). Sequencing of 16S ribosomal RNA (16S rRNA) from the cecal contents of broilers revealed that the composition of the chicken gut microbiota was altered by LST supplementation. The α-diversity of microbiota in broilers was increased (*p* < 0.05) in the LST1 group, but was decreased (*p* < 0.05) in the LST2 and LST4 groups compared with the LST0 group. The differential genera enriched in the LST1 group, such as *Bacillus, Odoribacter, Sutterella, Anaerofilum, Peptococcus*, were closely related to the increased growth performance, antioxidant status, intestinal morphology, Ca, Mg, and reduced blood lipid in the treated broilers.

## Introduction

For several decades, the use of antibiotic growth promoters (AGPs) in the livestock sector enhanced growth performance, intestinal health, feed efficiency, and mitigation of the stress caused by environmental and management factors in intensive poultry farming (1). However, with the long-term and extensive use of AGP, public concerns have augmented with respect to resistant bacteria and antibiotic drug residues (2). For this, many countries such as the European Union (3) and, recently, China have completely banned the utilize of AGP as growth promoters in animal diets. Given the high demand for high-quality poultry products, the development of sustainable, safe, practical, and effective feed additives that can both maintain broiler health and enhance productive potential is imperative. Presently, the use of herbal plants and their derivatives is gaining more attention globally for poultry production that could support in maintaining health status and growth performance (4, 5). Development of abundant plant resource for use as feedstuff or additives is one of the most important strategies to support sustainable animal production.

*Lasia spinosa* Thw. (LST) is a perennial herb, which grows extensively in numerous moist areas in tropical and subtropical regions (6). LST contains high content of fiber and a range of health-enhancing molecules such as flavonoids, phenolic compounds, and carotenoids, which have antioxidant, anti-inflammatory, and antihyperlipidemic properties; therefore, it has been used as a medicine in folk for many years to cure various diseases such as cholic, intestinal disorders, and rheumatism and even humans in the Bodo communities used LST as the cheapest source of nutrition to supplement vitamins and minerals (7), representing beneficial potency in poultry diets.

The functional properties of LST have already been applied to animal production. In some earlier studies, dietary inclusion of LST meal has been described to have boosted the performance production and feed efficiency in fish and buffalo (8, 9). This may attribute to the improvement of intestinal health in fish by LST supplementation (8). Based on the evidences stressing out a significant ability of LST to promote growth performance of animals, we designed this study to evaluate the effects of LST dietary inclusion on the growth performance, blood metabolites, antioxidant profile, intestinal morphology, and cecal microbiota in broilers, aiming at increasing knowledge and providing innovative information on the usage of LST in chicken feed as a brand new natural additive to support the sustainable and better development of livestock industry.

## Materials and Methods

This study was conducted on the Experiment Farm of Guangxi University and the Guangxi Fufeng Agriculture and Animal Husbandry Corporation, Ltd (Nanning, PR China). The bird maintenance and experimental protocols in this study were conducted following the guidelines approved by the Institutional Animal Care and Use Committee of Guangxi University (GXU2018-003).

### Preparation of the Plant

The rhizomes of LST were collected from Chongzuo, PR China. The fresh plants were washed, sliced into flake, and dried in the open air without sunlight, at a temperature of around 30°C for a week. The dry rhizomes of LST, a total of 11.5 kg, were machined into powder and then stored at room temperature (28°C) until use.

### Bird and Experimental Design

A total of 400 1-day-old chickens (males, Guangxi partridge) were individually weighed [initial body weight (BW): 42.52 ± 0.06 g] and randomly assigned into four experimental groups. Refer to the grouping method of the previous study (8), the experimental groups were as the following: (1) LST0 group, a basal diet; (2) LST1 group, a basal diet with 1% LST powder; (3) LST2 group, a basal diet with 2% LST powder; and (4) LST4 group, a basal diet with 4% LST powder. The LST powder was added into feed and mixed manually. Each experimental group was replicated in 10 cages of 10 chickens with a homogeneous initial body weight (IBM) per cage (70 cm-width × 70 cm-length × 35 cm-height). Food and water were automatically delivered *ad libitum* during the whole tested period. The composition of the basal diet is shown in Table 1. All the nutrients met or exceeded the Nutritional Research Committee (NRC) (1994) recommendations. Furthermore, ingredients and nutrient contents of LST were detected by Ulanqab E-more Tech Corporation Ltd., Wulanchabu, Inner Mongolia, and were shown in Table 2.

**Table 1:** Ingredients and nutrient contents of basal diet of growing broilers.

**Item**	**1–21 d**	**22–42 d**
**Ingredients (%)**		
Maize 8.5%	57	59.8
Soybean meal 44%	26.6	22.3
Wheat bran	3	3
Fish meal	2	6.6
Wheat middling	2.7	2.5
Barley	3	1.6
Limestone	1.5	1.6
Calcium hydrogen phosphate III type	1.2	1.5
Health product (SENERBO)	1	0.7
Premix^a^	2	2
Total	100	100
**Calculated analysis^b^**		
Metabolizable energy (MJ/kg)	11.93	12.35
Crude protein (%)	21	19.5
Lysine (%)	1.15	1.08
Methionine (%)	0.42	0.4
Calcium (%)	0.96	0.94
Available phosphorus (%)	0.59	0.55
Sodium chloride (%)	0.51	0.5
Crude fiber (%)	2.5	2.4
Crude ash (%)	4.8	4.7

a *Provides per kg of diet: vitamin A, 12,000 IU; vitamin D3, 5,000 IU; vitamin E, 130.0 mg; vitamin K3, 3.605 mg; vitamin B1 (thiamin), 3.0 mg; vitamin B2 (riboflavin), 8.0 mg; vitamin B6, 4.950 mg; vitamin B12, 17.0 mg; niacin, 60.0 mg; D-biotin, 200.0 mg; calcium D-pantothenate, 18.333 mg; folic acid, 2.083 mg; manganese, 100.0 mg; iron, 80.0 mg; zinc, 80.0 mg; copper, 8.0 mg; iodine, 2.0 mg; cobalt, 500.0 mg; and selenium, 150.0 mg*.

b *Calculated according to NRC (10)*.

**Table 2:** Ingredients and nutrient contents of *Lasia spinosa* Thw. (LST).

**Components (%)**	**As sampled**	**Dry matter basis**
Moisture (%)	12.20	-
Dry matter (%)	87.80	-
Crude protein (%)	7.02	8.0
Acid detergent fiber (%)	22.39	25.5
Neutral detergent fiber (%)	27.52	31.34
Lignin (%)	2.81	3.20
Non-fiber carbohydrate (%)	41.31	47.05
Starch (%)	22.13	25,2
Fat (%)	0.09	0.10
Ash (%)	11.86	13.51
Calcium (%)	1.44	1.64
Phosphorus (%)	0.43	0.49
Magnesium (%)	0.32	0.37
Potassium (%)	1.73	1.97
Sulfur (%)	0.35	0.40
Chloride (%)	1.19	1.36

All the chickens were maintained in an environmentally controlled chicken house. The birds were subjected to a 24-h photoperiod in the first 3 days and then hours of lights were progressively reduced to 10L:14D. The room temperature was retained at 36°C for the first week and then it was decreased by 4°C weekly and at the 5–6 weeks it was maintained at 22°C. Besides, all the animals were vaccinated with a Newcastle disease vaccine (LaSota Strain, 1,000 chicks/bottle, YEBIO Bioengineering Corporation Ltd. of Qingdao, Qingdao, PR China) by intranasal immunization on the 1st and 10th day and a deactivated infectious bursal disease vaccine (B87 Strain, 1,000 chicks/bottle, YEBIO Bioengineering Corporation Ltd. of Qingdao, Qingdao, PR China) by oral administration on 21st day.

### Growth Performance

Chickens were individually weighed at 21 and 42 days of age to define the average daily gain (ADG). Feed intake (FI) of chickens in each replicate was monitored daily and feed conversion ratio (FCR) (g feed/g gain) was considered by using data of ADG and FI. The mortality rate was recorded daily in each group. All the growth parameters were considered for the periods from day 1 to day 21, from day 22 to day 42, and from day 1 to day 42 according to the study protocol.

### Sampling Collection

At the end (day 42) of the experiment, chickens were deprived of feed for 12 h. Then, 40 chickens (1 chicken per cage) were selected on treatment-wise average live weight to be representative and slaughtered by cervical dislocation to collect a blood sample. The blood samples were separated from serum by centrifugation at 4°C and 3,000 rpm for 10 min. Serums were transferred into 1.5 ml microcentrifuge tubes and finally stored at −80°C for further examination. Immediately after slaughter, all the segments of the small intestine, as well as the cecum, were excised.

The gastrointestinal contents were collected from the cecum, hired in cryogenic vials, kept on dry ice, and delivered to the laboratory and stored at −80°C. Cecal contents were used for the detection of microbiota composition. For the sample of histological examination, 3 cm of the small intestinal segment was taken from the middle of the duodenum, jejunum, ileum, and cecum, respectively, washed in phosphate-buffered saline (PBS) solution to eliminate all the content, and fixed in 10% neutral buffered formalin solution (Solarbio, Beijing, PR China). For the assessment of the antioxidants in liver and breast muscles, tissues were collected into 1.5 ml Eppendorf tubes, then washed by PBS and transported to the laboratory on dry ice, and frozen immediately in liquid nitrogen (−196°C).

### Blood Metabolites Assessments

Serum biochemical metabolites, including liver function indices, kidney function indices, ionic content, glucose, rheumatoid factor (RF), lipids, and lipoproteins, were assessed. Serum aspartate aminotransferase (AST), alanine aminotransferase (ALT), uric acid (UA), total protein (TP), albumin (ALB), direct bilirubin (DB), total bilirubin (TB) urea, cholesterol (CHOL), RF, triglyceride (TG), high-density lipoprotein cholesterol (HDL-C), and low-density lipoprotein cholesterol (LDL-C) levels were determined spectrophotometrically using commercial kits (URIT Medical Electronic Corporation Ltd., Guilin, PR China). All the assays were conducted by the URIT-8021AVet Auto-Blood Biochemical Analyzer (URIT Medical Electronic Corporation Ltd., Guilin, PR China) according to the instructions of the manufacturer. Serum ionic levels, including calcium (Ca), phosphorus (P), iron (Fe), and magnesium (Mg), were evaluated using an atomic absorption spectrophotometer (URIT Medical Electronic Corporation Ltd., Guilin, PR China).

### Antioxidant Parameters Analysis

Approximately, 0.1 g of tissue samples (liver and breast muscle) were collected and homogenized with 0.9 ml PBS (0.01 mol/l), pH 7.4 using a Tissue Grinder Homogenizer (Tiangen Biotech Corporation, Beijing, PR China). Afterward, the supernatants were collected after centrifugation at 3,000 g for 20 min at 4°C. The levels of malondialdehyde (MDA), reactive oxygen species (ROS), catalase (CAT), superoxide dismutase (SOD), and glutathione peroxidase (GSH-Px) in tissue (liver and breast muscle) samples and the serum as well as the levels of TP in tissue samples (breast muscle and liver) were evaluated using ELISA technique (Shanghai Enzyme-linked Biotechnology Corporation Ltd., Shanghai, PR China, 96-well plates) with a microplate reader according to the instructions of the manufacturer (Shanghai Enzyme-linked Biotechnology Corporation Ltd., Shanghai, PR China). All the data were normalized alongside TP level in each tissue sample for intersample assessment.

### Histomorphology Study

The fixed tissues were dehydrated and fixed with paraffin in a 2T-12M tissue processor (Xiaogan Yaguang Medical Electronic Technology Corporation Ltd., Xiaogan, PR China) and then embedded in paraffin blocks using an embedding system (Leica, Germany, UK). The blocks were then sliced into 7 μm thick sections using a rotary microtome (Leica, Germany, UK) and stained with H&E. The crypt depth (Cd) (from the basis of the villus to the submucosa), the villus height (Vh) (from the tip of the villus to the crypt), and the Vh/Cd ratio were evaluated (11). Histomorphology analyses were performed on 6 well-oriented and intact villi and 6 crypts chosen from the duodenum, jejunum, ileum, and cecum. Slices were photographed and measured using an EVOS™ M5000 Imaging System (Thermo Fisher Scientific, Waltham, Massachusetts, USA).

### Microbiota Characterization

Cecal contents (five replicates/treatment) were collected and sent to Beijing Microread Technology Corporation Ltd. (Beijing, PR China) for 16S sequencing using an Illumina HiSeq 2500 PE 250 platform (Illumina, San Diego, California, USA). All the sequence data processing were performed using the Quantitative Insights Into Microbial Ecology (QIIME) software package (version 1.9.1). Sequences were paired-end and aligned against the GreenGenes database (http://greengenes.lbl.gov). The Mothur software package (version 1.23.1) was used to identify and remove chimeric sequences. Operational taxonomic units (OTUs) were assigned at a 97% identity using the GreenGenes database. Finally, the Venn diagram with shared and unique OTUs was used to identify the similarity and differences among treatments. α- and β-diversity analyses were performed for differences in species composition among samples. α-diversity analysis consisting of community diversity (Shannon and Simpson) and richness (phylogenetic diversity (PD) whole tree, Chao 1, and observed species) was performed using the Mothur software package. The microbiota were compared for β-diversity using the distance matrices generated from weighted UniFrac analysis, principal component analysis (PCA), and similarity analysis (ANOMIS) analysis. Linear discriminant analysis (LDA) effect size (LEfSe) analysis was performed to identify differentially abundant bacterial taxa based on LDA score > 2.0. The Spearman's correlation analyses were conducted to identify the relationship between physicochemical factors and intestinal flora.

### Statistical Analysis

Statistical analysis was performed by the IBM SPSS Statistics version 23.0.0 software (IBM Incorporation, Chicago, Illinois, USA). All the data above were tested using the one-way ANOVA and the effect of dietary LST powder supplementation was evaluated using polynomial contrasts (linear and quadratic). Significant differences among the treatments were tested using the least significant difference (LSD) test. The results were expressed as the mean and SEM. *p* < 0.05 was considered as statistically significant. Means followed by different letters differed significantly (*p* < 0.05), followed by no or same letters indicated no significant difference.

## Results

### Growth Performance

Data on the growth performance of the broilers are shown in Table 3. Briefly, a significant increase (*p* < 0.01) of average daily feed intake (ADFI) during 22–42 days and a significant decrease (*p* < 0.01) of FCR during 1–42 days were observed in chickens with dietary addition of LST powder. Compared with the LST0 group, the ADG of chickens was significantly (*p* < 0.01) increased by dietary LST powder supplement during the overall periods. The highest values (*p* < 0.01) of BW at 42 days were noticed in the LST1 group, followed by both the LST2 and LST4 groups. Given BW at 21 days, no significant alterations were detected between the control and LST2 groups. The mortality rates were low in the control and tested groups (about 0.0–2.0% as a normal range) and were not influenced (*p* > 0.05) by dietary treatments.

**Table 3:** Effect of dietary LST powder supplementation on growth performance of broiler chickens.

**Item^1^**	**Treatment^2^**				**SEM^4^**	***P*** **-value^3^**		
	**LST0**	**LST1**	**LST2**	**LST4**		**ANOVA**	**Linear**	**Quadratic**
**Body weight (BW; g)**								
At one d	42.57	42.35	42.62	42.55	0.06	0.328	0.638	0.52
At 21 d	292.42^b^	321^a^	301.11^b^	318.29^a^	2.90	<0.01	0.008	0.219
At 42 d	712.90^c^	860.55^a^	771.81^b^	761.44^b^	9.96	<0.01	0.242	<0.01
**Average daily gain (ADG; g)**								
1–21 d	11.90^b^	13.27^a^	12.31^b^	13.13^a^	0.88	<0.01	0.008	0.215
22–42 d	20.02^c^	25.69^a^	22.41^b^	21.10^bc^	0.42	<0.01	0.985	<0.01
1–42 d	15.96^c^	19.48^a^	17.36^b^	17.12^b^	0.24	<0.01	0.244	<0.01
**Average Daily Feed Intake (ADFI; g)**								
1–21 d	24.15	24.88	24.67	25.37	1.11	0.092	0.026	0.962
22–42 d	49.91^b^	56.01^a^	50.63^b^	50.97^b^	0.60	<0.01	0.611	0.005
1–42 d	37.03^b^	40.45^a^	37.65^b^	38.17^b^	0.34	<0.01	0.801	0.013
**Feed Conversion Rate (FCR; g BW/g feed)**								
1–21 d	2.03^a^	1.88^c^	2.00^a^	1.93^b^	0.01	<0.01	0.037	0.024
22–42 d	2.51^a^	2.18^b^	2.26^b^	2.42^a^	0.03	<0.01	0.385	<0.01
1–42 d	2.27^a^	2.03^c^	2.14^b^	2.18^b^	0.18	<0.01	0.09	<0.01
**Mortality (%)**								
1–21 d	0	2.00	1.00	2.00	0.53	0.499	0.302	0.642
22–42 d	0	0	0	0	0	-	-	-
1–42 d	0	1	0.5	1	0.26	0.499	0.302	0.642

1 *BW, body weight; ADG, average daily gain; ADFI, average daily feed intake; FCR, feed conversion ratio*.

2 *LST, Lasia spinosa Thw.; LST0, control diet; LST1, 1% supplementation level of LST powder; LST2, 2% supplementation level of LST powder; LST4, 4% supplementation level of LST powder*.

3 *Mean values within a row with different letters differ at p < 0.05*.

4 *SEM*.

(a–c) *are the presented letters of statistical p-value. Same letters in a row means that p > 0.05, presenting the statistical results not significant difference. Different letters in a row means that p < 0.05, presenting the analysis results in significant difference*.

### Blood Metabolites

The blood metabolites and mineral profile of broilers fed with the different diets for 42 days were shown in Table 4. No significant differences were detected in all the blood metabolites, except for total bilirubin (TBIL), TG, HDL-C, and LDL-C. The levels of TBIL were significantly decreased (*p* < 0.01) in the LST1 group compared with other treated groups. The levels of TG and LDL-C were significantly reduced (*p* < 0.01) by dietary inclusion of LST, whereas the levels of HDL-C were significantly increased (*p* < 0.01) in all the supplemented groups. The mineral levels of Ca, Fe, and P were significantly increased (*p* < 0.01) in groups with dietary supplementation of LST. Only birds fed with 4% exhibited higher levels of magnesium (*p* = 0.003) compared with the other treated and control groups.

**Table 4:** Effect of dietary LST powder supplementation on blood metabolites and mineral profile of broiler chickens (*n* = 10).

**Item^1^**	**Treatment^2^**				**SEM^4^**	***P*** **-value^3^**		
	**LST0**	**LST1**	**LST2**	**LST4**		**ANOVA**	**Linear**	**Quadratic**
**Blood metabolites**								
ALT, U/L	2	2.1	2.1	2	0.08	0.946	1	0.548
AST, U/L	212.2	216.1	222	206.2	3.13	0.348	0.667	0.123
Total protein, g/L	37.83	39.22	38.27	39.28	0.64	0.831	0.569	0.887
Albumin, g/L	15.23	15.9	15.67	16.11	0.20	0.448	0.183	0.773
DBIL, μmol/L	3.76	3.63	3.79	3.79	0.05	0.583	0.556	0.494
TBIL, μmol/L	22.87^a^	14.68^b^	22.69^a^	21.97^a^	0.71	<0.01	0.221	<0.01
Urea, mmol/L	0.85	0.90	0.82	0.85	0.02	0.357	0.529	0.768
Uric Acid, μmol/L	106	114.9	105.3	112.8	6.15	0.933	0.851	0.956
Triglyceride, mmol/L	0.65^a^	0.467^b^	0.523^b^	0.512^b^	0.02	0.006	0.032	0.021
Cholesterol, mmol/L	4.20	4.37	4.36	4.13	0.08	0.64	0.776	0.211
HDL_C, mmol/L	1.42^b^	1.66^a^	1.6^a^	1.625^a^	0.02	<0.01	<0.01	0.002
LDL_C, mmol/L	1.60^a^	0.84^d^	1.42^b^	1.13^c^	0.06	<0.01	0.005	<0.01
Glucose, mmol/L	10.56	10.31	10.11	10.47	0.18	0.837	0.779	0.421
RF, IU/mL	3.45	3.42	3.79	3.55	0.09	0.448	0.401	0.555
**Mineral profile**								
Calcium, mmol/L	7.07^d^	7.80^a^	7.38^c^	7.61^b^	0.05	<0.01	<0.01	<0.01
Iron, μmol/L	15.91^c^	17.9^b^	18.88^ab^	19.63^a^	0.35	<0.01	<0.01	0.268
Magnesium, mmol/L	0.87^b^	0.85^b^	0.91^b^	0.98^a^	0.01	0.003	<0.01	0.066
Phosphorus, mmol/L	2.10^c^	2.31^b^	2.39^b^	2.56^a^	0.03	<0.01	<0.01	0.682

1 *ALT, alanine aminotransferase; AST, aspartate aminotransferase; DBIL, direct bilirubin; TBIL, total bilirubin; HDL-C, high-density lipoprotein cholesterol; LDL-C, low-density lipoprotein cholesterol; RF, rheumatoid factor*.

2 *LST, Lasia spinosa Thw.; LST0, control diet; LST1, 1% supplementation level of LST powder; LST2, 2% supplementation level of LST powder; LST4, 4% supplementation level of LST powder*.

3 *Mean values within a row with different letters differ at p < 0.05*.

4 *SEM*.

(a–d) *are the presented letters of statistical p-value. Same letters in a row means that p > 0.05, presenting the statistical results not significant difference. Different letters in a row means that p < 0.05, presenting the analysis results in (a–c) are the presented letters of statistical p-value. Same letters in a row means that p > 0.05, presenting the statistical results not significant difference. Different letters in a row means that p < 0.05, presenting the analysis results in significant difference*.

### Antioxidant Status

The antioxidant profiles of serum, liver, and breast muscle on broilers are given in Table 5. Except for the levels of SOD and GSH-Px in liver (*p* > 0.05), the levels of SOD, CAT, and GSH-Px in serum, liver, and breast muscle showed a significantly (*p* < 0.05) linear response to LST powder addition level, while the addition of LST powder linearly decreased the concentrations of ROS and MDA in serum, liver, and breast muscle (*p* < 0.05).

**Table 5:** Effect of dietary LST powder supplementation on serum, liver, and muscle antioxidant status of broiler chickens (*n* = 6).

**Item^1^**	**Treatment^2^**				**SEM^4^**	***P*** **-value^3^**		
	**LST0**	**LST1**	**LST2**	**LST4**		**ANOVA**	**Linear**	**Quadratic**
**Serum**								
ROS, IU/mL	468.28^a^	449.93^a^	347.53^b^	363.49^b^	16.91	0.011	0.003	0.542
MDA, nmol/mL	8.13^a^	7.52^ab^	6.42^b^	6.95^b^	0.22	0.027	0.013	0.147
SOD, ng/mL	3.52^b^	4.52^a^	4.60^a^	5.35^a^	0.21	0.01	0.001	0.731
CAT, pg/mL	354.02^c^	448.06^b^	511.58^a^	495.54^ab^	15.07	<0.01	<0.01	0.004
GSH-Px, ng/mL	149.52^b^	167.36^ab^	183.40^a^	185.50^a^	4.27	0.003	<0.01	0.239
**Liver**								
ROS, IU/mg.pro	787.18^a^	618.71^b^	601.94^b^	440.50^c^	34.03	0.001	<0.01	0.942
MDA, nmol/mg.pro	14.47^a^	11.71^b^	10.96^bc^	9.17^c^	0.51	<0.01	<0.01	0.478
SOD, ng/mg.pro	14.71	14.37	13.46	14.36	0.64	0.925	0.748	0.654
CAT, pg/mg.pro	844.19^b^	1214.18^a^	1275.68^a^	1351.24^a^	52.36	<0.01	<0.01	0.051
GSH-Px, ng/mg.pro	361.38	387.06	417.96	425.84	11.61	0.18	0.035	0.691
**Breast muscle**								
ROS, IU/mg.pro	798.37^a^	663.31^ab^	535.79^b^	638.09^b^	30.22	0.011	0.012	0.026
MDA, nmol/mg.pro	14.1^a^	12.64^ab^	9.98^c^	11.04^bc^	0.49	0.008	0.003	0.127
SOD, ng/mg.pro	10.83^b^	13.56^a^	15.23^a^	13.18^a^	0.49	0.007	0.022	0.006
CAT, pg/mg.pro	726.99^b^	1049.43^a^	1167.52^a^	1164.84^a^	50.98	0.001	<0.01	0.04
GSH-Px, ng/mg.pro	304.27^b^	398.56^a^	390.45^a^	380.55^a^	12.05	0.01	0.021	0.015

1 *ROS, reactive oxygen species; MDA, malondialdehyde; SOD, superoxide dismutase; CAT, catalase; GSH-Px, glutathione peroxidase*.

2 *LST, Lasia spinosa Thw.; LST0, control diet; LST1, 1% supplementation level of LST powder; LST2, 2% supplementation level of LST powder; LST4, 4% supplementation level of LST powder*.

3 *Mean values within a row with different letters differ at p < 0.05*.

4 *SEM*.

(a–c) *are the presented letters of statistical p-value. Same letters in a row means that p > 0.05, presenting the statistical results not significant difference. Different letters in a row means that p < 0.05, presenting the analysis results in (a–c) are the presented letters of statistical p-value. Same letters in a row means that p > 0.05, presenting the statistical results not significant difference. Different letters in a row means that p < 0.05, presenting the analysis results in significant difference*.

### Histomorphology Study

**Figure 1:**
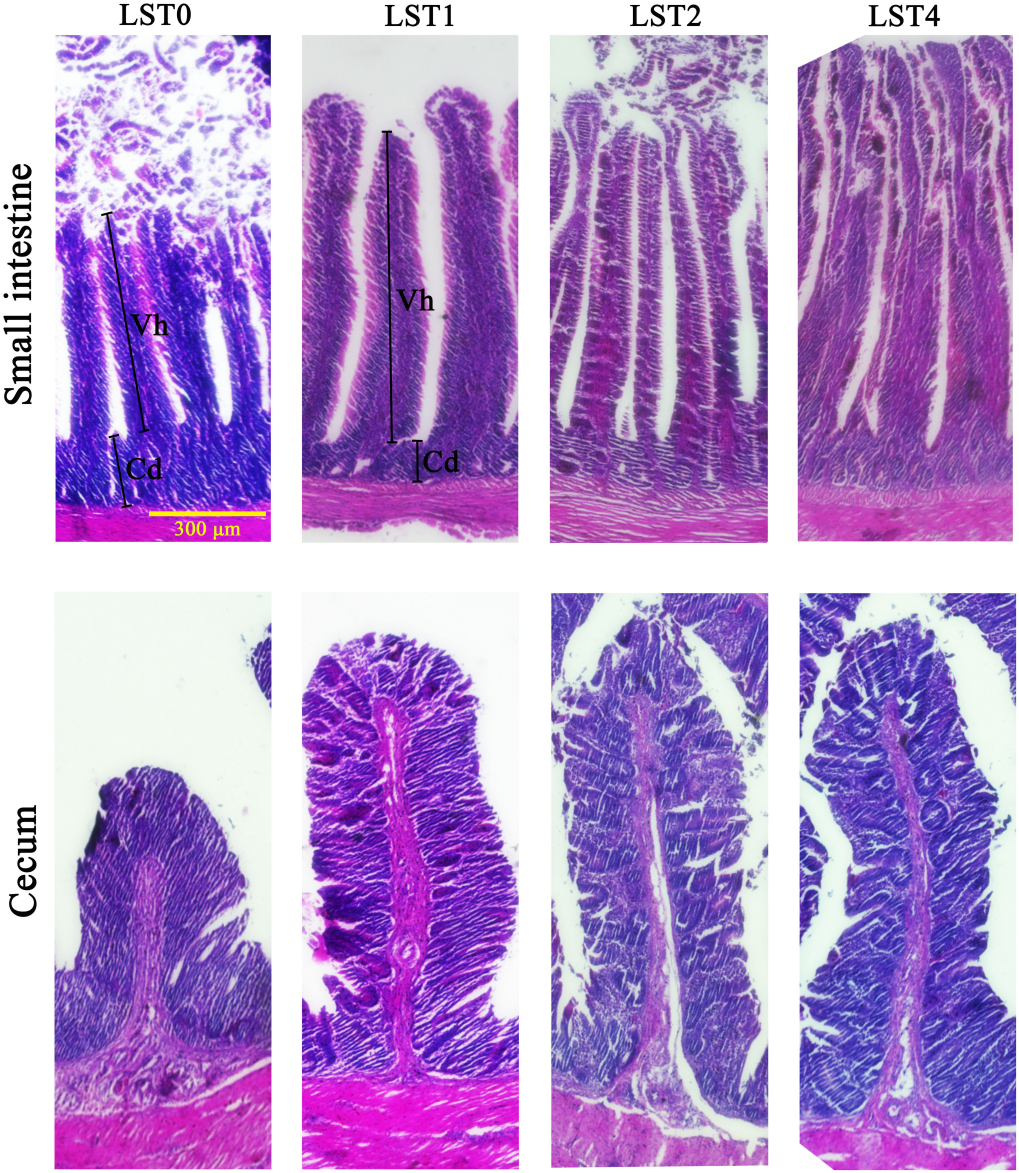
Histomorphology of chicken small intestine (ileum) and cecum. LST, *Lasia spinosa* Thw.; LST0, control diet; LST1, 1% supplementation level of LST powder; LST2, 2% supplementation level of LST powder; LST4, 4% supplementation level of LST powder; Vh, villus height; Cd, crypt depth. Bar = 300 μm.

Table 6, Figure 1 represent the data with respect to the histological features of broilers fed with test diets for 42 days. Birds fed with different levels of LST showed greater (*p* < 0.05) Vh (*p* < 0.01) and Vh/Cd of the intestinal and cecum compared with the control group. On the other hand, the Cd was significantly (*p* < 0.05) decreased in the intestinal and cecum of all the LST-treated groups as compared with the control group.

**Table 6:** Effect of dietary LST powder supplementation on the morphology of the small intestine and cecum of broiler chickens (*n* = 10).

**Item^1^**	**Treatment^2^**				**SEM^4^**	***P*** **-value^3^**		
	**LST0**	**LST1**	**LST2**	**LST4**		**ANOVA**	**Linear**	**Quadratic**
**Duodenum**								
Vh, μm	1173.62^b^	1528.74^a^	1503.23^a^	1524.74^a^	36.69	<0.01	<0.01	0.007
Cd, μm	236.77^a^	180.25^b^	185.67^b^	224.09^ab^	8.71	0.045	0.656	0.006
Vh/Cd	5.09^b^	9.06^a^	8.56^a^	7.35^a^	0.42	0.002	0.055	0.001
**Jejunum**								
Vh, μm	1020.47^c^	1263.61^b^	1381.79^b^	1536.02^a^	38.31	<0.01	<0.01	0.372
Cd, μm	215.42^a^	149.14^b^	188.62^ab^	175.26^ab^	8.63	0.046	0.268	0.109
Vh/Cd	5.19^b^	8.56^a^	7.75^a^	9.49^a^	0.40	<0.01	<0.01	0.211
**Ileum**								
Vh, μm	692.73^c^	1042.04^b^	1069.29^b^	1295.16^a^	43.66	<0.01	<0.01	0.275
Cd, μm	144.19^ab^	116.15^b^	133.05^b^	169.00^a^	6.15	0.011	0.065	0.005
Vh/Cd	5.18^b^	9.27^a^	8.42^a^	8.06^a^	0.41	0.001	0.014	0.002
**Cecum**								
Vh, μm	757.09^c^	933.04^b^	983.65^ab^	1126.84^a^	32.19	<0.01	<0.01	0.748
Cd, μm	158.21^ab^	118.48^b^	151.38^ab^	206.27^a^	11.27	0.045	0.068	0.031
Vh/Cd	5.04^b^	8.06^a^	7.36^a^	6.51^ab^	0.39	0.034	0.258	0.012

1 *Vh, villus height; Cd, crypt depth; Vh/Cd, villus height to crypt depth ratio*.

2 *LST, Lasia spinosa Thw.; LST0, control diet; LST1, 1% supplementation level of LST powder; LST2, 2% supplementation level of LST powder; LST4, 4% supplementation level of LST powder*.

3 *Mean values within a row with different letters differ at p < 0.05*.

4 *SEM*.

(a–c) *are the presented letters of statistical p-value. Same letters in a row means that p > 0.05, presenting the statistical results not significant difference. Different letters in a row means that p < 0.05, presenting the analysis results in (a–c) are the presented letters of statistical p-value. Same letters in a row means that p > 0.05, presenting the statistical results not significant difference. Different letters in a row means that p < 0.05, presenting the analysis results in significant difference*.

### Microbiota Characterization

**Figure 2:**
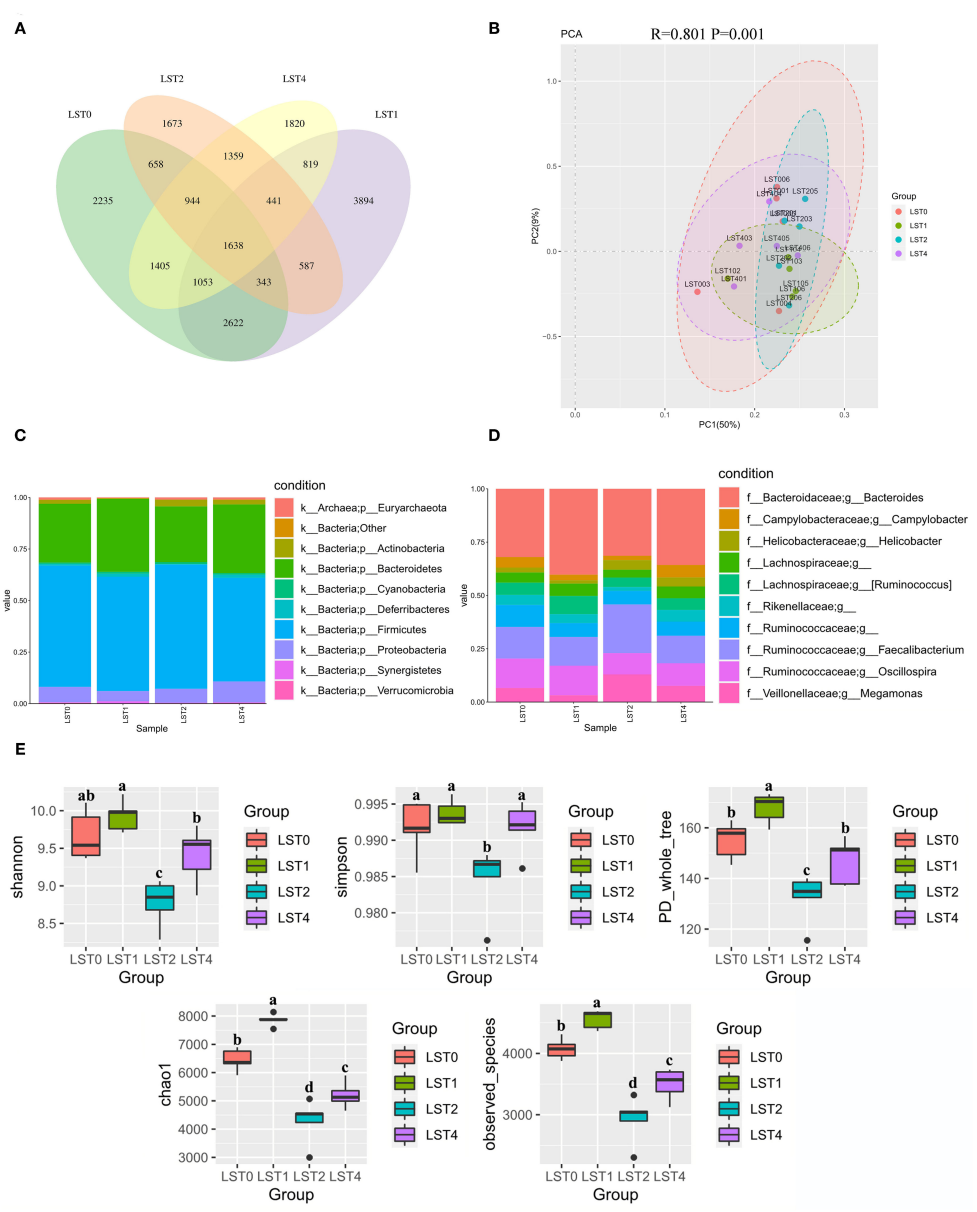
Abundance and diversity of microbial community in cecal contents of broiler chickens after dietary supplementation of LST powder for 42 days. **(A)** The Venn diagram summarizing the numbers of common and unique OTUs in the microflora community. **(B)** The PCA plot about the cecal microflora. **(C,D)** Microbial composition at the phylum and genus level. **(E)** α-diversity about the cecal microbial community. ^a−*d*^Boxes followed by different letters differ significantly (*p* < 0.05), followed by no or same letters indicated no significant difference (*p* > 0.05). OTUs, operational taxanomic units; PCA, principal component analysis; LST, *Lasia spinosa* Thw.; LST0, control diet; LST1, 1% supplementation level of LST powder; LST2, 2% supplementation level of LST powder; LST4, 4% supplementation level of LST powder.

The Venn diagram revealed that the cecal microbiome of broilers shared 1,638 OTUs across the groups (Figure 2A). The unique OTUs were 2,235, 3,894, 1,673, and 1,820 in the LST0, LST1, LST2, and LST4 groups, respectively. The results of PCA plot demonstrated that a significant distinction among the control and LST treatment groups was identified (*p* = 0.001), suggesting that the intestinal microbiota was significantly altered by LST supplementation (Figure 2B). In addition, the composition of the top 10 phyla and genera in cecal contents of broilers were detected (Figures 2C,D). Based on this analysis, the dominant phyla of cecal microbiota were *Firmicutes, Bacteroidetes, Proteobacteria, Verrucomicrobia, Synergistetes, Deferribacteres, Cyanobacteria, Actinobacteria*, and *Euryarchaeota* (Figure 2C). At the genus field, the dominant microbiome in cecal contents was *Bacteroides, Faecalibacterium, Oscillospira, Megamonas, Helicobacter, Campylobacter*, and *Ruminococcus* (Figure 2D).

PD whole-tree index, Chao1 index, and observed species index revealed that the α-diversity was significantly higher (*p* < 0.05) in the LST1 group compared with the control group. In contrast, α-diversity in the LST2 and LST4 groups was significantly reduced (*p* < 0.05) compared with other groups. These results suggest that the gut microbiota of broiler was significantly altered by dietary LST supplementation (Figure 2E).

**Figure 3:**
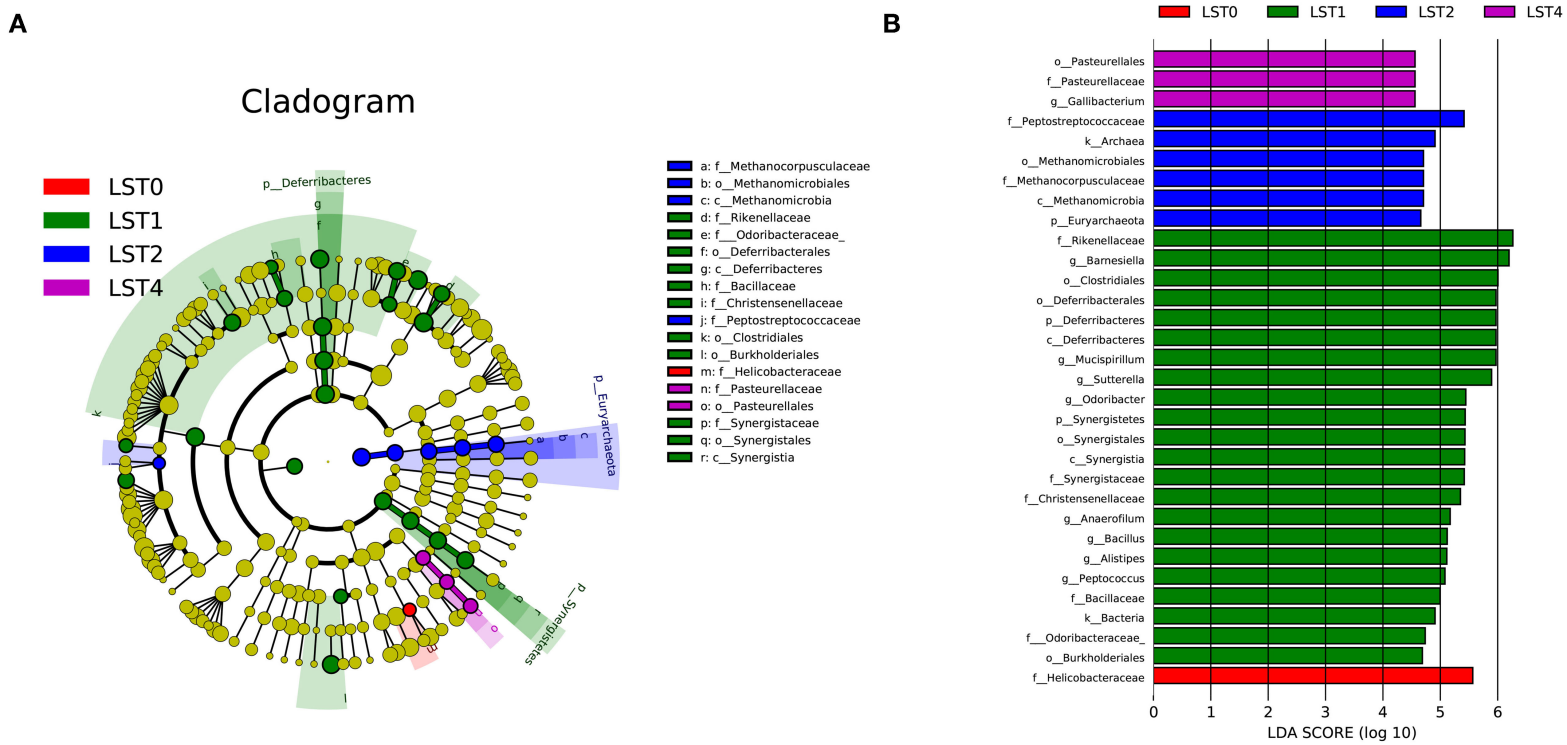
Linear discriminant analysis (LDA) effect size (LEfSe) of cecal microbiota in broilers fed with LST powder at day 42. **(A)** Phylogenetic profile of specific bacterial taxa and predominant bacteria among the four different groups, as determined using the LEfSe analysis. Biomarker taxa are heighted by colored circles and shaded areas. Each diameter of circle is relative to abundance of taxa in the community. **(B)** Histogram of LDA score calculated for differential abundant taxa with cutoff LDA score > 2.0. LST, *Lasia spinosa* Thw.; LST0, control diet; LST1, 1% supplementation level of LST powder; LST2, 2% supplementation level of LST powder; LST4, 4% supplementation level of LST powder.

Linear discriminant analysis effect size analysis was performed to identify specific phylotypes of the microbiome as responsive to LST supplemented into broiler diets at 42 days (Figure 3). As shown in Figure 3A, a total of 18 bacterial taxonomic clades showed statistical differences among all the test diets. At the class level, birds fed with 1% LST had a greater (*p* < 0.05) abundance of *Deferribacterales* and *Synergistales* than those fed with the other test diets. At the order level, *Deferribacterales, Clostridiales, Burkholderiales*, and *Synergistales* were overrepresented (*p* < 0.05) in the LST1 group, while *Methanomicrobiales* and *Pasteurellales* were overrepresented (*p* < 0.05) in the LST2 and LST4 groups, respectively. In addition, at the family level, the relative abundances of *Rikenellaceae, Odoribacteraceae, Bacillaceae, Christensenellaceae*, and *Synergistaceae* were significantly increased (*p* < 0.05) in the LST1 group compared with those in other groups. The abundances of *Helicobacteraceae, Methanocorpusculaceae, Peptostreptococcaceae*, and *Pasteurellaceae* were significantly increased (*p* < 0.05) in the LST0, LST2, and LST4 groups, respectively. LDA score calculated for differential abundant taxa is shown in Figure 3B. Totally, at the genus level, *Barnesiella, Mucispirillum, Sutterella, Odoribacter, Anaerofilum, Bacillus, Alistipes*, and *Peptococcus* were enriched (*p* < 0.05) in the LST1 group, while only *Gallibacterium* was enriched (*p* < 0.05) in the LST4 group. The results further indicated that the dietary LST supplementation altered the abundance of gut microbiota compared with the control group.

### Correlation Analysis of Gut Microbiota With Biological Pathways

The Spearman's correlation analysis of biological pathways was conducted to better understand the regulatory function of the gut microbiome as affected by the inclusion of dietary LST. Association of differential genera with growth performance, serum parameters, antioxidant status, and intestinal morphology were recognized (Table 7). The relative abundance of *Alistipes* was positively related to ROS of breast muscle, duodenum Cd, and triglyceride, while negatively correlated with serum and hepatic CAT level and Vh/Cd of the duodenum. The relative abundance of *Barnesiella* was positively related to serum ROS and MDA, while negatively correlated with hepatic GSH-Px. Additionally, the abundance of *Mucispirillum* was positively related to Ca and jejunal Vh/Cd, while negatively correlated with jejunal Cd. The abundance of *Bacillus* was positively related to serum CAT and SOD, Ca, duodenal Vh and Vh/Cd, SOD, and GSH-Px of breast muscle, while negatively correlated with FCR, TBIL, and LDL-C at 1–42 days. The abundance of *Peptococcus* was positively related to Ca, while negatively correlated with TBIL and LDL-C. Furthermore, the relative abundance of *Gallibacterium* was positively related to Mg and LDL-C, while negatively correlated with serum ROS contents. A positive correlation was observed between *Odoribacter* and ADG and BW at 1–42 days, while *Sutterella* was positively related to Mg level in the serum.

**Table 7:** The differential genera that were separately correlated with growth performance, blood metabolites, antioxidant status, and intestinal morphology in 42-day-old broiler chickens by using the Spearman's correlation analyses (*n* = 5).

**Genus**	**Performance^1^**	**Correlation coefficient**	***P*-value**
Alistipes	CAT (serum)	−0.491729323	0.028
Alistipes	Triglyceride	0.449718312	0.047
Alistipes	CAT (liver)	−0.469172932	0.037
Alistipes	Duodenum Vh/Cd	−0.454135338	0.044
Alistipes	Duodenum Cd	0.481203008	0.032
Alistipes	ROS (breast muscle)	0.461654135	0.04
Barnesiella	ROS (serum)	0.491729323	0.028
Barnesiella	MDA (serum)	0.552295115	0.012
Barnesiella	GSH-Px (liver)	−0.568421053	0.009
Mucispirillum	Ca	0.518448942	0.019
Mucispirillum	Jejunum Cd	−0.562617565	0.01
Mucispirillum	Jejunum Vh/Cd	0.516735651	0.02
Bacillus	CAT (serum)	0.518689418	0.019
Bacillus	SOD (serum)	0.598530933	0.005
Bacillus	F: G (1–42 days)	−0.46666282	0.038
Bacillus	TBIL	−0.580175398	0.007
Bacillus	LDL-C	−0.596165198	0.006
Bacillus	Ca	0.531902155	0.016
Bacillus	Duodenum Vh	0.625895742	0.003
Bacillus	Duodenum Vh/Cd	0.499770655	0.025
Bacillus	SOD (breast muscle)	0.611706669	0.004
Bacillus	GSH-Px (breast muscle)	0.65585045	0.002
Peptococcus	TBIL	−0.456999133	0.043
Peptococcus	LDL-C	−0.450993047	0.046
Peptococcus	Ca	0.562620313	0.01
Anaerofilum	CAT (serum)	−0.481203008	0.032
Sutterella	Mg	0.446032375	0.049
Gallibacterium	ROS (serum)	−0.498499154	0.025
Gallibacterium	LDL-C	0.486560546	0.03
Gallibacterium	Mg	0.464581954	0.039
Gallibacterium	Cecum Cd	0.478801619	0.033
Odoribacter	ADG (1–42 days)	0.479699248	0.032
Odoribacter	42 d BW	0.479699248	0.032

1 *ADG, average daily gain; FCR, feed conversion ratio; TBIL, total bilirubin; LDL-C, low-density lipoprotein cholesterol; ROS, reactive oxygen species; MDA, malondialdehyde; SOD, superoxide dismutase; CAT, catalase; GSH-Px, glutathione peroxidase; Vh, villus height; Cd, crypt depth; Vh/Cd, villus height to crypt depth ratio*.

## Discussion

In the last decades, dietary manipulations with innovative feed supplements, such as medicinal plants and their derivatives, are among the common advanced strategies (3). Medicinal plants are reflected as a cornerstone of medicine since earliest times and have been employed as promoters of antioxidant capacity, immunity, and growth in animals (12). LST has been used medicinally since ancient times for its diverse biological activities such as antibacterial and antiseptic properties (7).

In this study, dietary LST powder supplementation significantly boosted the growth performance of the chickens involved, produced a remarkable increase in ADG and final BW, and caused a statistical decrease in FCR when compared to the control diet. These results are in accordance with the findings that the inclusion of LST in fish diets significantly improved the growth performance and feed utilization (8). In addition, this study displayed no significant difference in mortality rate was noticed in groups that received LST compared with the control, which agreed with a study by Munglue et al. (8) who perceived no significant alterations concerning survival rate in fish as a response to the dietary LST. In parallel, the supplementation of LST in buffalo had a positive impact on the growth indices (9). However, the underlying mechanisms of the growth-promoting effect of LST are still not understood. Phesatcha et al. (13) found that the inferior FCR and boosted growth may relate to enhanced appetite and digestion in beef cattle fed with diets enriched with LST (60–90 g/day/head). The authors suggested that this could be related to the constructive role of LST in improving the FI, apparent digestibility, and efficiency of microbial N synthesis in cattle (13). Similarly, Abolfathi et al. (14) found that elecampane (*Inula helenium* L.) rhizome showed positive effects on growth performance and this improvement in growth parameters was accompanied by significant increase in apparent ileal digestibility of dry matter, organic matter, ether extract, and gross energy. Thus, the improved digestibility may be one of the motives in this study.

As a natural additive in animal feed, several phytohormones found in LST are proven to promote growth and feed utilization. Suthikrai et al. (9) found that LST contains considerable levels of natural phytoestrogens (10.76–14.55 pg/g DM) and phytoandrogens (0.15–0.92 pg/g DM). Therefore, it is reasonable to hypothesize that the production of growth hormone and insulin-like growth factor-1 may be modulated by phytohormones, resulting in the improvement of growth. Phytoandrogens may also enhance protein synthesis by activating the androgen receptor (AR)-mediated anabolism (15). In addition, combined with the findings of Buszczak et al. (16), the growth-promoting effect of LST may be due to modulating related genes expression related to protein synthesis and cell growth. Moreover, LST is excessively rich in alkaloids, flavonoids, terpenoids, phenolic compounds, steroids, phytosterols, saponins, coumarins, tannins, glycosides, and anthraquinone (7). Kikusato et al. (17) found that plant-derived isoquinoline alkaloids supplementation significantly improved the growth performance of chickens under heat stress conditions. Starčević et al. (18) reported that phenolic compounds boosted the growth performance and antioxidant profile of chickens. It has been reported that phytosterols could promote animal production and antioxidant capacity (19). Thus, these phytochemical constituents may synergistically contribute to the growth-promoting property of LST. However, due to various factors such as localities, sample processing, and the methods of analysis, not only the levels of bioactive substances, but also the composition may be different.

Blood variables are commonly reliable indicators of general health profile, as responses to external and internal stressors and stimuli. This study notices that LST supplements did not significantly affect the levels of blood metabolites (except for TBIL, TG, HDL-C, and LDL-C), showing that LST had no detrimental impacts or toxicity in the broilers. In this study, dietary inclusion of LST reduced the TG and LDL-C and boosted TBIL and HDL-C levels in broiler serum. The findings were consistent with prior studies in rats where the levels of TG and LDL-C were significantly decreased with the methanolic leaves extract of LST (200, 400, and 800 mg/kg BW) administration in comparison to the control group (20). Moreover, Kaewamatawong et al. (21) studied the acute to subchronic toxicity of LST in mice and found that LST significantly decreased the TG values, but did not induce any toxicological effects in the acute and subchronic term. It was reported that flavonoids from corn silk exhibited strong regulatory activity on several serum lipid values, reducing the levels of TG and LDL-C and increasing the level of HDL-C (22). Furthermore, scientists presented new evidence supporting a key role for *Rhizoma coptidis* alkaloids as a regulator of liver–gut axis in hyperlipidemic mice (23). Therefore, the presence of antihyperlipidemic compounds such as flavonoids and alkaloids present in LST could be responsible for a decrease of the serum TGs and LDL-C and increase of the HDL-C levels and, thus, avoiding cardiovascular ailment (7). Further studies should be conducted to validate the underlined mechanism of LST as a natural lipid-lowering agent for drug treatments in poultry. This study has shown that LST significantly improved the levels of Ca, Fe, and P in broiler serum as a response to 4% addition of LST in broiler diets. This enhancement could be due to the rich trace element content in LST (7). In addition, TBIL was significantly reduced only in the LST1 group and other biochemical parameters were similar among the groups, thus indicating that the LST powder consumption did not have a negative influence on the health status of the animals.

In the physiological process, free radicals and other oxygen-derived species are continuously produced *in vivo*, some of which can cause serious damage to biological molecules, especially to DNA, lipids, and proteins. Lipid peroxidation is a fundamental cellular degenerating process caused by free radicals and readily occurs in the tissues rich in highly oxidizable polyunsaturated fatty acids, which may contribute to cell death and disease in living organisms (24). The elimination of free radicals depends on the action of several enzymes such as SOD, GSH-Px, and CAT (25). Moreover, chicken meat is relatively rich in polyunsaturated fatty acids, making it extremely vulnerable to oxidation (26). Therefore, diet-derived antioxidants may be vital in reducing cumulative oxidative damage. Natural antioxidants are broadly found in medicinal herbs. 5 g/kg supplementation of ginger root powder enhanced the oxidative stability of broilers (27). *Pulicaria gnaphalodes* powder in broiler diets significantly increased the SOD and GSH-Px activities and decreased the MDA level in serum, liver, and thigh muscle (28). According to a large amount of literature, plant polyphenolic flavonoids was one of the major groups of compounds contributing mainly to the total antioxidant activity (29). The LST and its extracts have antioxidant properties due to its high content of flavonoids and phenolic compounds (7). It has been reported that the extracts of LST could scavenge the stable radical 1,1-diphenyl-2-picrylhydrazyl (DPPH), elucidating those polyphenols and ascorbic acid acted mainly as the primary antioxidant free-radical terminators of rhizomes (30). Besides, phytochemical constituents in LST such as flavonoids and phytosterols (19, 31) were proven to improve the antioxidant enzyme activity.

In this study, we measured the levels of antioxidant enzymes to determine the antioxidant status in serum, liver, and breast muscle. We found that GSH-Px, SOD, and CAT were significantly increased in the LST supplementation groups, while the levels of ROS and MDA, as indicators of OS, were significantly reduced in the LST supplementation groups. The increased antioxidant enzyme content is parallel with the decreased level of MDA and ROS in response to dietary LST inclusion, indicating that dietary LST supplements can promote the antioxidant capability of broilers by enhancing the enzymatic antioxidant scheme. As the improvement of the antioxidant enzyme would exert a positive influence on meat quality (19), so it is logical to speculate that the addition of LST can enhance the meat quality of the chickens involved in this experiment.

Results of this study indicated that the dietary LST powder addition affected the structure of the intestine in broilers. It is well-known that intestinal morphology can objectively reflect gut health and Vh, as well as Cd, can partially reflect the functional status of the intestine (11). A previous study demonstrated that the Vh was increased and the intestinal function was improved in fish after supplementation of LST extract in diet (8). Similarly, in this study, a statistical decrease in Cd and a statistical increase in Vh and the ratio of the two were observed in the intestine of chickens fed with LST powder compared with the control group. The increased Vh and Vh/Cd ratio for various gut fractions of chickens fed with LST powder were in accordance with appropriate growth performance and increased metabolizability of nutrients. In fact, the increase of Vh usually results in a higher total luminal villus absorptive area and subsequently leads to higher transport of nutrients at the villus surface (32). Moreover, the decrease in Cd usually indicates a slower turnover and healthier condition of the gut, which can reduce maintenance requirements and ultimately be beneficial to promote the growth of the animals (33). Rich fiber fractions in LST rhizome (7) may sustain the intestinal integrity through increasing the Vh and Vh/Cd and decreasing the Cd feature (34).

The gut microbiome is closely related to intestinal health, normal physiological functions, and poultry production and the composition of the microbial community could be altered upon the diet and over time (35). This study is setup necessarily to evaluate the effect of LST powder as a feed additive on the intestinal flora of Guangxi partridge broilers, which will contribute to developing nutrition intervention for optimal health, growth, and productivity in poultry. We found that the dominant phyla in the chicken are *Firmicutes, Bacteroidetes*, and *Proteobacteria*. Furthermore, the primary genera were *Bacteroides, Faecalibacterium*, and *Oscillospira* in this study, which was consistent with earlier works recognizing *Bacteroidetes* as the fundamental genus in the cecum (about 40% of sequences) (36).

Dietary LST powder addition also caused a significant alteration of the gut microbiota diversity in broilers. According to the α-diversity results, the 1% supplementation of LST powder significantly increased the abundance of caecal microbiota (PD whole-tree index, Chao1 index, observed species, and unique OTUs) compared with the other groups, while 2 and 4% supplementation of LST powder decreased the abundance (Chao1 index and observed species) of cecal microbiota. In addition, with respect to the β-diversity analysis, a clear separation of microbial community due to dietary LST powder inclusion was observed, which means microbial communities of each group were significantly different. Elevated levels of diversity mostly contribute to maintaining the stability of intestinal microbiota after exposure to environmental stress as well as resistance against potential invading pathogens (37). For these reasons, it is considered that a reduction of α- and β-diversity should be negative. This suggests that the structure of intestinal flora in the LST1 group was improved and, thus, better intestinal health. However, the LST2 and LST4 groups got a lower diversity and this may be due to the antibacterial activity of higher LST powder addition (7). However, no antibacterial substances were specified in these studies (7) and further investigation is required for isolating the specific bioactive constituents responsible for antibacterial properties.

Linear discriminant analysis effect size analysis revealed that the relative abundance of *Anaerofilum* was significantly increased in the LST1 group compared with other groups, which has been proposed to boost the antioxidant capability and absorption of energy as previously reported by Guo et al. (38). Interestingly, *Odoribacter* (39, 40) and *Sutterella* (41), known as short-chain fatty acid (SCFA)-producing bacteria, were also overrepresented in the LST1 group. SCFAs, as the main energy source of colorectal cells, play an essential role in regulating the absorption of several nutrients, digestive and hormonal secretions in the intestine, and participating in energy metabolism widely (39, 41). Further investigation will be focused on measuring the composition and levels of SCFAs in broilers supplemented with LST powder. Notably, *Sutterella*, a potential probiotic, could contribute to improving the growth rate and the feed conversion ratio of chickens (42) and its deficiency might lead to the destruction of the normal function of colonic epithelium and the induction of inflammatory bowel disease, suggesting its vital role in maintaining intestinal health (43). In addition, *Bacillus* has been widely proven to promote growth performance (41, 44). In this study, we found the enrichment of *Bacillus* in the LST1 group, while the best growth performance was found in the LST1 group. Therefore, the increase of relative abundance in *Anaerofilum, Odoribacter, Sutterella*, and *Bacillus* in the LST1 groups in this study suggests the efficacy of 1% LST powder in promoting beneficial bacteria, which would eventually contribute to improved performance and health. These findings suggest that there is a close association between intestinal flora and physiological performance.

However, no reports have verified the associations of growth performance, serum metabolites, antioxidant status, and intestinal morphology with changed gut microbiome structure in Guangxi partridge broilers fed with LST powder. By using the Spearman's correlation analysis, we revealed that the profiles of taxonomic composition at the genus level in cecal microbiota were significantly associated with growth performance, serum metabolites, antioxidant status, and intestinal morphology. Consistently, the relative abundance of *Odoribacter* and *Bacillus* was positively correlated with growth performance, which showed that 1% addition level of LST powder could improve ADG and decrease FCR potentially by increasing the abundance of *Odoribacter* and *Bacillus* as detected at day 42. In this study, we also demonstrated that *Bacillus*, which has been reported to improve intestinal morphology (45, 46), was positively related to the Vh and Vh/Cd of the duodenum. Furthermore, the abundance of *Bacillus* was closely correlated to the concentrations of CAT, SOD, and GSH-Px and many studies have reported that *Bacillus* could participate in boosting of the antioxidant scheme (47–49). Actually, intestinal flora can regulate the well-being, immunity, and disease of host by determining the biological value of the diet (50). In this study, we found that serum metabolites including TG, TBIL, Ca, Mg, and LDL-C affected by dietary treatment of LST powder were faithfully connected with the dynamic fluctuations of broiler gut microbiome structure.

Overall, the diversity of gut microbiota is reshaped by LST supplements and further contributes to the improvement of growth performance, intestinal morphology, antioxidant capacity, Ca and Mg, and reduction of TG, LDL-C, and TBIL.

## Conclusion

Our results indicate that LST powder contributed to the improvement of growth performance, intestinal morphology, antioxidant capacity, Ca, Mg, Fe and P, and reduction of blood lipids. Especially, 1% supplementation of LST significantly increased the diversity of gut microbiota, which further promoted physiological performance. Of note, during the antibiotic-free era, LST has a great potential application in broiler feeding when the importance of nutrition, feed processing, management, and biosafety strategies is highly recognized.

## Data Availability Statement

The datasets presented in this study can be found in online repositories. The names of the repository/repositories and accession number(s) can be found in the article/Supplementary Material.

## Ethics Statement

The animal study was reviewed and approved by the Institutional Animal Care and Use Committee of Guangxi University.

## Author Contributions

YH and LZ contribute to the investigation, formal analysis, and writing—original draft. YLi contributes to the project administration. KT and HS contribute to the investigation. XL contributes to the writing—review and editing. YT contributes to the resources. AH and SA contribute to the writing—original draft. WS, KS, and TT contribute to the conceptualization. YLu contributes to the writing—review and editing and funding acquisition. All authors contributed to the article and approved the submitted version.

## Funding

This study was jointly supported by the National Natural Science Foundation of China (No. 31960157), the Guangxi Pioneer of Animal Science and Technology (No. 202109-02), and the Guangxi Special Fund for Invited Expert.

## Conflict of Interest

YT was employed by Guangxi Fufeng Agriculture and Animal Husbandry Corporation Ltd. The remaining authors declare that the research was conducted in the absence of any commercial or financial relationships that could be construed as a potential conflict of interest.

## Publisher's Note

All claims expressed in this article are solely those of the authors and do not necessarily represent those of their affiliated organizations, or those of the publisher, the editors and the reviewers. Any product that may be evaluated in this article, or claim that may be made by its manufacturer, is not guaranteed or endorsed by the publisher.
